# Practices of vitamin D supplementation leading to vitamin D toxicity: Experience from a Low-Middle Income Country

**DOI:** 10.1016/j.amsu.2021.103227

**Published:** 2022-01-05

**Authors:** Siraj Muneer, Imran Siddiqui, Hafsa Majid, Nawazish Zehra, Lena Jafri, Aysha Habib Khan

**Affiliations:** Department of Pathology and Laboratory Medicine, Aga Khan University, Stadium Road, P.O. Box 3500, Karachi, 74800, Pakistan

**Keywords:** VDT, Vitamin D toxicity, 25OHD, 25-hydroxyvitamin D, VD, Vitamin D, VDD, Vitamin D deﬁciency, IM, IntramuscularI, Vitamin D, Supplements, Hypervitaminosis D, Toxicity, Deficiency, Adults, Children, Pakistan

## Abstract

**Introduction:**

The trend of prescribing VD preparations for nonspecific body aches and self-medication has increased significantly. The importance of vitamin D toxicity (VDT) has been underestimated and under recognized. This study was done to determine the frequency toxicity (>150 ng/ml) in subjects for 25-hydroxyvitamin D (25OHD) and evaluate the vitamin D (VD) supplements used by these subjects.

**Methodology:**

This descriptive cross-sectional study was conducted at the Section of Chemical Pathology, Aga Khan University Hospital Karachi from April 2020 to March 2021. Subjects with 25OHD toxicity were contacted and information related to history of calcium and VD supplementation were collected. The statistical analysis was performed using the Microsoft Excel 2016.

**Results:**

Over a year period 105398 subjects were tested for serum 25OHD, of which 0.34% (n = 364) subjects had 25OHD level of >150 ng/ml. After satisfying exclusion criteria 186 subjects (78 were <18 years of age and 108 were adults) were included in final analysis. All of these were using VD supplements and the main indications were delayed growth/short height (43.7%, n = 34) and aches or pains in (54.6%, n = 59) in pediatric and adult subjects respectively.

Most of the subjects were taking supplements orally (74.1%, n = 138). Commonly prescribed preparation in adults and pediatric was 200,000 IU (70.4%, n = 76) and 400 IU (35.9%, n = 28) respectively. Most subjects took supplements for 1–3 months (68.3%, n = 127). Stated total supplementation ranged from 20,000 IU to 3600,000 IU in pediatric subjects and 200,000 IU to 96,00,000 IU in adults.

**Conclusions:**

Supplementation is a leading cause of potential toxic levels of 25OHD. The condition can be prevented by careful use of VD supplements and consistent monitoring.

## Highlights

• Vitamin D toxicity is extremely rare; but it may happen at high doses. The use of vitamin D supplementation should be guarded and monitored.
• In this study we observed that vitamin D toxicity (>150 ng/ml) was prevalent, present in 0.34% (n=364) subjects.
• Main indications of supplementation were delayed growth in children and aches or pains in adults

## 1 Introduction

High prevalence of vitamin D deﬁciency (VDD) is well recognized in Pakistan and globally [1]. Strategies and recommendations to combat VDD have been put forward but there is wide variation and no consensus on any one strategy [2,3]. Hence, Practice patterns of physicians in treating VDD vary due to lack of clear guidelines on optimal dosing regimen and availability of different vitamin D (VD) preparations. Replacement of VD for maintaining sufficient bone health is necessary but achieving balance between optimal and toxic levels is equally important. For this serum 25-hydroxy VD (25OHD) testing is recommended to adjust dose accordingly. International Osteoporosis Foundation recommends measuring serum 25OHD levels after 2–3 months of VD replacement (3). However very few physicians perform biochemical testing to assess the status of 25OHD prior to or after VD replacement [4].

The trend of prescribing VD preparations for nonspecific body aches in VD deficient endemic areas and self-medication with over the counter VD supplements has increased significantly [5,6]. According to studies, serum 25OHD levels above 150 ng/ml are considered toxic levels [[7], [8], [9]]. Self and prescribed supplementation or errors of labeling of VD formulations, or inadvertent use such administration of high doses of VD in infants or children for complaints such as delayed teething, ‘late walking’, and ‘knock-kneed gait’ are reported [10].

The importance of vitamin D toxicity (VDT) has been underestimated and under recognized. This study was done to determine the frequency of subjects identified with 25OHD toxicity (>150 ng/ml) and evaluate the VD supplements used by these subjects.

## 2 Material and methods

This prospective cross-sectional study was conducted at the Section of Chemical Pathology, Department of Pathology and Laboratory Medicine, AKUH Karachi Pakistan after approval from Aga Khan University Hospital's Ethics Review Committee (ERC ID: 2019-1973-6924). Data of subjects tested for serum 25OHD between April 2020 to March 2021 was reviewed daily and those with 25OHD levels >150 ng/ml were contacted via telephone. Only the initial results of the subjects tested at Clinical Laboratory were included. Those with incomplete clinical history, 25OHD levels <150 ng/ml, whose contact numbers were unavailable or not answering telephone calls were excluded. To maintain confidentiality all identifiers were removed, and study identity numbers were generated. The work has been reported in line with the STROCSS criteria [11].

After explaining the reason for phone call, verbal informed consent was taken and clinical information of calcium and VD supplementation including prescribed or self–medicated, indications for prescription, formulation strength, total dosage, frequency, duration, and calcium status was collected on a structured clinical history forms. The hypercalcemia was labeled when serum calcium was >10.2 mg/dl.

## 3 Biochemical analysis

Serum 25OHD levels were analyzed on Liaison immunoassay analyzer (DiaSorin Inc. Diagnostics, USA) using chemiluminescence immunoassay technique. The assay's analytical measurement range was 4.0–150 ng/ml. Two-level quality control materials were run with each batch of samples for internal quality control. External proficiency was assured by analyzing samples from the College of American Pathologists thrice per year, throughout the study period, and all external proficiency surveys during the study period were acceptable. The following reference ranges were used and relate to 25OHD levels: Deficient <20 ng/mL, insufficient 21–29 ng/mL, sufficient/adequate 30–100 ng/mL, high or hypervitaminosis D > 100 ng/mL, toxicity >150 ng/mL (2, 8).

## 4 Data analysis

The statistical analysis was performed using the Microsoft Excel 2016. Subjects were categorized into two age groups: <18 years (pediatric) and ≥18 years (adult). Total doses of VD supplements were added to calculate cumulative supplementation and daily supplementation was calculated by dividing cumulative dose by duration of supplementation.

Descriptive statistics median (interquartile range, IQR) were calculated for numerical data while frequency (percentage) for categorical data. Frequencies of subjects with VD toxicity were derived and their correlates were evaluated in the both the age groups. Demographics (age and gender), calcium status of subjects, indications, formulation strengths, frequency, duration, cumulative and daily dose of supplementation were generated.

## 5 Results

**Fig. 1:**
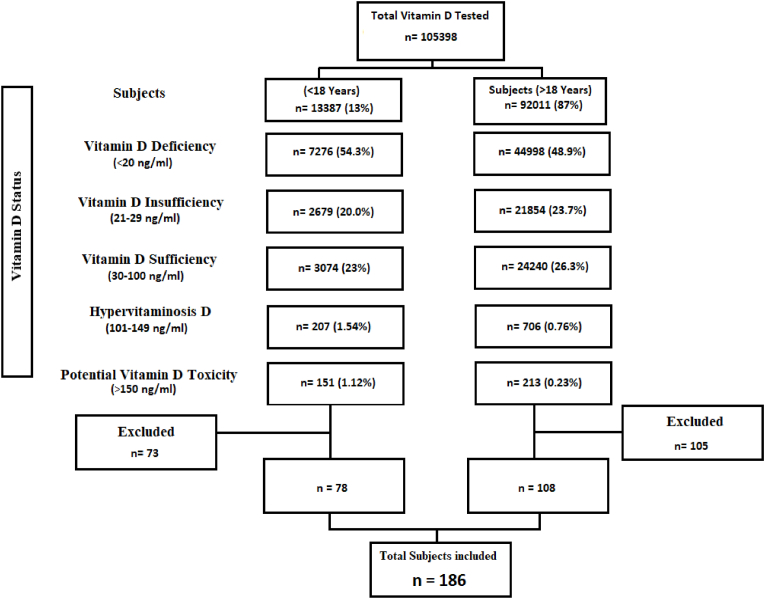
Consort showing distribution of subjects tested for VD from April 2020 to March 2021 at AKUH clinical laboratories.

During one-year period 105,398 subjects were tested for serum 25OHD levels at Clinical Laboratories. Fig. 1 shows the distribution of subjects tested for 25OHD. Toxicity was observed in 0.34% (n = 364) subjects. Of these 186 subjects (78 pediatric and 108 adults) were included in final analysis with 63% (n = 117) being female.

In 90% (n = 167) subjects [92% (n = 72) pediatric and 88% (n = 95) adults] VD supplement was prescribed by physician. Table 1 shows the demographics, indications for testing and available calcium status of the subjects. The main indications for taking VD supplements in pediatric population were delayed growth/short height (43.7%m, n = 34), VDD (24.4%, n = 19) and delayed walking/milestones (19.2%, n = 15). In contrast, the main indications in adults were aches/pains (54.6%, n = 59) and VDD in (32.4%, n = 35) of subjects. Serum calcium levels were available for 34% (n = 63) subjects with median calcium levels of 9.75 (0.9) mg/dl.

**Table 1:** Characteristics of Pediatric (<18 years) and Adult (>18 years) subjects tested at AKUH clinical laboratories with indications of VD supplementation.

		<18 Years n = 78	>18 Years n = 108
**Demographics**	Male	36 (46.2%)	33 (30.6%)
	Female	42 (53.8%)	75 (69.4%)
	Age (Years)	2.0 (3.9)	41.4 (27.4)
**Indications for VD Supplementation**	VD Deficiency (<20 ng/dl) n (%)	19 (24.4%)	35 (32.4%)
	Bone Pain n (%)	3 (3.8%)	59 (54.6%)
	Delayed Growth/Short Height n (%)	34 (43.7%)	–
	Delayed Walking/Milestones n (%)	15 (19.2%)	–
	Renal insufficiency n (%)	–	6 (5.6%)
	Bowing of Legs n (%)	3 (3.8%)	–
	COVID = 19 n (%)	–	4 (3.7%)
	Others n (%)	4 (5.1%)	4 (3.7%)
**Calcium Status of Subjects**	Normal Calcium levels	20 (76.9%)	26 (70.3%)
	Calcium (mg/dl)	9.6 (0.5)	9.7 (0.5)
	Hypercalcemia	6 (23.1%)	11 (29.7%)
	Calcium (mg/dl)	10.9 (0.32)	12.1 (2.5)

IQR= Interquartile range, VD= Vitamin D, COVID-19 = Coronavirus Disease 2019, Median (IQR) was reported for age, and calcium levels.

Table 2 shows details on VD supplementation regarding dose, formulation types, route and duration. Majority subjects used oral supplements with different formulation strengths of VD ranging from 400 IU to 600,000 IU. In adults, 70.6% (n = 76) were taking 200,000 IU oral, 14.8% (n = 16) took 600,000 IU, intramuscularly. While in pediatric subjects 35.9% (n = 28) were taking 400 IU/drop, (ranging from 1 to 10 drops per day) and 11.5% (n = 9) subjects were taking 200,000 IU/ml (6,666 IU/drop) and number of drops ranged from 1 to 2 drops per day. Stated total supplementation ranged from 20,000 IU to 3600,000 IU in pediatric subjects, which was calculated to range from 400 IU to 28,571 IU/day. While 200,000 IU to 96, 00,000 IU in adults is calculated to range from 2,000 IU to 200,000 IU (Table 2). Duration of supplementation varied from 1 week to years with majority taking supplementation for 2–3 months (Table 2).

**Table 2:** Details of VD supplementation in subjects with 25 (OH)D levels >150 ng/ml.

			<18 Years n = 78	>18 Years n = 108
**Cumulative and daily supplementation**	**Total Cumulative VD Supplements taken (I.U) Median (IQR)**		4,20,000 (600,000)	16,00,000 (14,00,000)
	**VD Supplements Taken per day Median (IQR)**		12,066 (18,300)	27,066 (15,238)
**VD supplementation route**	**Oral**		64 (82.1%)	74 (68.5%)
	**Intramuscular**		10 (12.8%)	24 (22.2%)
	**Both (Oral & IM)**		4 (5.1%)	10 (9.3%)
**VD supplementation strengths used (IU)**	**400/drop**		28 (35.9%)	–
	**6,666/drop**		9 (11.5%)	–
	**5,000**		–	1 (0.9%)
	**10,000**		–	1 (0.9%)
	**20,000**		–	2 (1.9%)
	**40,000**		–	1 (0.9%)
	**50,000**		–	5 (4.6%)
	**200,000**		24 (30.8%)	76 (70.4%)
	**600,000**		5 (6.5%)	16 (14.8%)
	**Combination**		12 (15.3%)	6 (5.6%)
	**200,000 +**	**400**	8 (10.3%)	–
	**600,000+**	**400**	3 (3.8%)	–
		**6666**	1 (1.2%)	–
		**10,000**	–	1 (0.9%)
		**50,000**	–	2 (1.9%)
		**200,000**	–	3 (2.8%)
**Duration of VD supplementation**	**Once**		6 (7.7%)	2 (1.8%)
	**1 month**		22 (28.2%)	14 (12.9%)
	**2**–**3 months**		33 (42.3%)	58 (53.8%)
	**4**–**6 months**		8 (10.3%)	15 (13.9%)
	**7**–**12 months**		3 (3.8%)	6 (5.6%)
	**>1 year**		6 (7.7%)	13 (12%)

IQR= Interquartile range, VD= Vitamin D, IM= Intramuscular.

## 6 Discussion

In 1970s, VD was central to eradication of rickets and osteomalacia. However, identification of vitamin D receptors in many organs and tissues in the body linked VDD to a variety of outcomes. Together with greater public interest and increased cognizance about benefits of VD resulted in VD becoming a popular supplement and one of the most common over the counter medicines nowadays [12]. Many of the supplements are in the form of unregulated formulations dispensed with little guidance for safe administration. VD is not a benign drug and excessive supplementation can lead to serious complications and hospitalization. Khan MN et al. evaluated the causes of hypercalcemia other than solid tumour malignancy and found out that 17.3% (n = 25) patients had hypercalcemia due to VDT [13]. A paradigm shift from VDD to a global increase in cases of VDT is been noted. A decade-long observational on data (unpublished) from our laboratory showed increasing number of individuals with elevated serum 25OHD levels with increase in number of request for 25OHD testing. The prevalence of potential VDT in our study was 1.12% in pediatric subjects and 0.23% adult subjects (Fig. 1).

Contextual guidelines on appropriate VD replacement are lacking, leading to varied prescribing practices among physicians, aggressive replacement of VD and increased risk of VDT [13,14]. The Institute of Medicine's report and The Endocrine Society guidelines in 2011 suggested that higher levels of 2000, 4000 and 10000 IU/day for infants, children and adults respectively may be needed to correct VDD [2,15]. However, in this study median daily dose was much higher than the recommended dose in both pediatrics age group (12,066 IU/day) and adults (27,066 IU/day). Caglar A et al. cited similar findings in 38 hospitalized children diagnosed with VDT during a 5-year study period [16]. All children were taking vitamin D supplements with the median dose of 1,200,000 IU. Nine of these used supplements for delayed teething and late walking. Misgar RA et al. reported VDT in 32 adults with hypercalcemia in 3-year prospective study [14]. The main indication for supplementation were aches and pain (n = 22) and none of the patients had prior 25OHD testing neither was subjected to biochemical monitoring. Similarly, the diagnosis of rickets, osteomalacia, osteoporosis or VDD by serum 25OHD level testing was also not determined in most of the subjects in the present study prior to prescribing VD.

In the present study, although most of the subjects had an initial prescription from the physician. It is likely that the key components of a prescription like formulation strength, dosage, duration and follow-up were either not properly highlighted in prescription or not followed by subjects. This is evidenced by the increased dosage (3–10 drops) taken by a 22% (n = 17) children out of the total 28 children who were prescribed 1–2 VD drops/day. Out of these 17 children, three developed hypercalcemia. Bilbao et al. described similar cases of two infants who developed hypercalcemia after receiving over-the-counter vitamin D supplementation far above the recommended dose [17].

In this study, it was identified that large doses of supplements were continuously taken for months and years without monitoring of serum 25 (OH) D levels in contrast to the recommendations by Endocrine society to monitor 25OHD levels after 2–3 months of prescribing VD supplements (Supplemental Table 1 a & b). An adult subject was taking 5000 IU/day for more than 5 years without monitoring of 25OHD levels. Another subject took intramuscular (IM) injections of 200,000 IU on alternate days for 2 weeks along with 200,000 oral capsules once a week for 5 years. A pediatric subject was taking 200,000 IU oral supplements once a week for more than a year. and details are mentioned in Supplemental Table 1 a & b.

In Pakistan, two preparations of 10 ml oral drops of VD are available, one with 400 IU/drop and other with 200,000 IU/ml or 6666 IU/drop (Counting 30 drops in the 1 ml dropper), which is 16-fold higher than 400 IU. Nine (11.5%) children were receiving two drops daily (cumulative dose 13,332 daily) and two of them developed hypercalcemia.

Raj Kumar K et al. reported similar case of dosing error with over-the-counter VD supplement in a 3 month-old Asian-American infant who was prescribed 400 IU once daily [18]. Initially parents were administering 1 mL daily of 400 IU/mL infant vitamin D solution. But after sometime they replaced 400 IU/mL infant vitamin D solution with a new brand of infant vitamin D liquid preparation which provided 400 IU of vitamin D per drop. Without noticing the difference in the concentrations between the two preparations, the parents were giving the infant 1 mL daily of the new preparation. So the infant was receiving a 30-fold overdose of vitamin D (12,000 IU) daily. In our study, one pediatric subject accidently took 10 ml bottle of VD drops (400IU/drop) in one go and developed hypercalcemia.

The VDT has a varied presentation ranging from asymptomatic to acute life threatening complications mainly due to hypercalcemia. In our study, eleven adults developed hypercalcemia {median calcium levels 10.7 mg/dl (0.2)}, out of these six were hospitalized. Pandita KK et al. reported 15 cases of hypervitaminosis D with hypercalcemia, 12 had evidence of renal dysfunction with median creatinine level of 2.1 mg/dl [19]. Genzen JR et al. reported 25OHD levels of >150 ng/ml in [n = 15 (∼0.03%)] and [n = 27 (∼0.05%)] patients with hypercalcemia in 4 and 2 patients from Weill Cornell Medical College/New York Presbyterian Hospital and University of Iowa Hospitals and Clinics respectively [20]. Masood et al. in a prospective intervention study in ambulatory care setting, 100 subjects with VDD were randomized to receive a dose of 600,000 or 200,000 IU of Cholecalciferol via an oral or IM route and serum 25OHD measures at 2, 4 and 6 months after the intervention showed that VDD was corrected in 70.6–93.85% of participants and the mean levels remained significantly higher from baseline in all groups at all-time points during the 6 months of observation [21]. Basit et al. used a single IM dose of 600,000 IU of vitamin D3 for the treatment of painful diabetic neuropathy and reported significant increase in vitamin D and calcium levels in patients at 20 weeks [22].

## 7 Conclusion

We investigated a large cohort of subjects with serum 25OHD levels of >150 ng/ml. Our study demonstrates approach of physicians and public towards VD supplementation. There is a rising trend of prescribing VD preparations for nonspecific body aches in this part of the world. Health effects of toxicity can be serious and need prompt diagnosis. The condition can only be prevented by spreading awareness among healthcare providers regarding the toxic potential of high doses of VD, careful use of VD supplements and consistent monitoring of people during supplementation. The effects of different dosing regimens have not been studied in our setting and practices are based on individual experiences. However, at the same time, there is a strong need to have our own guidelines regarding dosages and dosing interval for correction of VDD as there are no published guidelines from Pakistan with the available preparations.

## Ethical approval

The study protocol was approved by Aga Khan University Hospital's Ethics Review Committee (ERC ID: 2019-1973-6924, Date: 15.12.2019).

## Patients’ consent

Verbal informed consents were obtained from all patients.

## Availability of data and materials

The data set used in the current study are available from the corresponding author upon reasonable request.

## Funding

This project was not funded.

## Authors’ contribution

SM: Conception and design of the work, data collection, analysis of data, led and conceived the project, and authored the manuscript, final approval and agreement.IS: Design of the work, analysis of data, discussion, final approval and agreement.HM: Design, Collecting and analyzing data, discussion, final approval and agreement. NZ: Design, Collecting and analyzing data, final approval and agreement. LJ: Design, statistics and analyzing data, discussion, final approval and agreement.

## Provenance and peer review

Not commissioned, externally peer-reviewed.

## Annals of medicine and surgery

The following information is required for submission. Please note that failure to respond to these questions/statements will mean your submission will be returned. If you have nothing to declare in any of these categories then this should be stated.

## Please state any sources of funding for your research

This project was not funded.

## Consent

Verbal informed consent was taken.

## Registration of research studies

1. Name of the registry: Clinicaltrials.gov
2. Unique Identifying number or registration ID: NCT05139576
3. Hyperlink to your specific registration (must be publicly accessible and will be checked): https://clinicaltrials.gov/ct2/show/results/NCT05139576?term=NCT05139576&draw=2&rank=1

## Guarantor

Dr. Hafsa Majid.

## Declaration of competing interest

The authors declared no conflict of interest.
