# Supplementary material for: Practices of vitamin D supplementation leading to vitamin D toxicity: Experience from a Low-Middle Income Country
Source: Ann Med Surg (Lond). 2022 Jan 5;73:103227. doi: 10.1016/j.amsu.2021.103227 (PMC8767303; doi:10.1016/j.amsu.2021.103227)
Supplement: Multimedia component 2 [file mmc2.docx]

**Table 3 (a): Details of VD supplementation strengths, dosing and duration in < 18 years old subjects with 25OHD levels of >150 ng/ml**  (**n=78**)

| **Strength (IU)** | Dose | **Duration** | **Subjects** |
| --- | --- | --- | --- |
| **400/drop**  **n= 28 (35.9%)** | One drop/d n= 2 (2.6%) | 6 months | 1 |
|  |  | 16 months | 1 |
|  | 2 Drops/d n= 8 (10.2%) | 1 months | 2 |
|  |  | 2 months | 2 |
|  |  | 3 months | 1 |
|  |  | 4 months | 1 |
|  |  | 8 months | 1 |
|  |  | >1 year | 1 |
|  | 3-4 Drops/d n= 9 (11.5%) | 1 months | 1 |
|  |  | 2 months | 1 |
|  |  | 3 months | 4 |
|  |  | 5 months | 1 |
|  |  | 8 months | 1 |
|  |  | >1 year | 1 |
|  | 5-6 Drops/d n= 3 (3.8%) | 2 months | 1 |
|  |  | 6 months | 2 |
|  | 8 drops/d n= 3 (3.8%) | 3 months | 1 |
|  |  | 6 months | 1 |
|  |  | 8 months | 1 |
|  | 10 drops/d n= 2 (2.6%) | 6 months | 1 |
|  |  | >1 year | 1 |
|  | 10ml bottle (accidental) n=1 (1.3%) | - | 1 |
| **6666/drop**  **n= 9 (11.5%)** | One drop n= 2 (2.6%) | 1 months | 2 |
|  | 2 Drops n= 7 (8.9%) | 1 months | 4 |
|  |  | 2 months | 2 |
|  |  | 3 months | 1 |
| **200,000**  **n= 24 (30.8%)** | Once n= 1 (1.3%) | - | 1 |
|  | On alternate days n= 1 (1.3%) | 5 weeks | 1 |
|  | Once a week n= 16 (20.5%) | 2 weeks | 3 |
|  |  | 3 weeks | 2 |
|  |  | 1 month | 3 |
|  |  | 5 weeks | 1 |
|  |  | 2 months | 4 |
|  |  | 3 months | 2 |
|  |  | >1 Year | 1 |
|  | Once in 15 days n= 5 (6.4%) | 1 month | 3 |
|  |  | 6 weeks | 1 |
|  |  | 3 months | 1 |
|  | Once in 2 month n= 1 (1.3%) | >1 year | 1 |
| **600,000**  **n= 5 (6.5%)** | Once n= 4 (5.1%) | - | 4 |
|  | Once in 6 weeks n= 1 (1.3%) | 3 months | 1 |
| **400 + 200,000 n= 1 (1.3%)** | 2 drops/d | 3 months | 1 |
|  | Once |  |  |
| **400 + 200,000 n= 1 (1.3%)** | 2 drops/d | 3 months | 2 |
|  | Once a week | 3 weeks |  |
| **400 + 200,000 n= 1 (1.3%)** | 2 drops/d | 1 month | 1 |
|  | Once in 15 days |  |  |
| **400 + 200,000 n= 1 (1.3%)** | 2 drops/d | 2 months | 1 |
|  | Once in 20 days |  |  |
| **400 + 200,000 n= 1 (1.3%)** | 6 drops/d | 1 month | 1 |
|  | Once |  |  |
| **400 + 200,000 n= 1 (1.3%)** | 3 drops/d | 3 months | 1 |
|  | Once a week |  |  |
| **400 + 200,000 n= 1 (1.3%)** | 2 drops/d | 3 months | 1 |
|  | Once in 15 days |  |  |
| **400 + 600,000 n= 1 (1.3%)** | 2 drops/d | 3 months | 1 |
|  | Twice |  |  |
| **400 + 600,000 n= 1 (1.3%)** | 2 drops/d | 6 months | 1 |
|  | Once |  |  |
| **400 + 600,000 n= 1 (1.3%)** | 2 drops/d | 2 months | 1 |
|  | Once |  |  |
| **6666 + 600,000 n= 1 (1.3%)** | One drop/d | 3 months | 1 |
|  | Once |  |  |
