# Supplementary material for: Practices of vitamin D supplementation leading to vitamin D toxicity: Experience from a Low-Middle Income Country
Source: Ann Med Surg (Lond). 2022 Jan 5;73:103227. doi: 10.1016/j.amsu.2021.103227 (PMC8767303; doi:10.1016/j.amsu.2021.103227)
Supplement: Multimedia component 3 [file mmc3.docx]

**Table 3 (b): Details of VD supplementation strengths, dosing and duration in > 18 years old subjects with 25OHD levels of >150 ng/ml** (**n=108**)

| **Strength (IU)** | **Dose** | **Duration** | **Subjects** |
| --- | --- | --- | --- |
| **5000 n= 1 (0.9%)** | Once daily n= 1 (0.9%) | 7-8 years | 1 |
| **10000 n= 1 (0.9%)** | Once daily n= 1 (0.9%) | 2 months | 1 |
| **20,000 I.U n= 2 (1.9%)** | Once daily n= 1 (0.9%) | 3 months | 1 |
|  | Once weekly n= 1 (0.9%) | 2 years | 1 |
| **40,000 n= 1 (0.9%)** | Once in 15 days n= 1 (0.9%) | 18 months | 1 |
| **50,000 n= 5 (4.6%)** | Once daily n= 1 (0.9%) | 1 month | 1 |
|  | Twice a week n= 1 (0.9%) | 3 months | 1 |
|  | Once a week n= 3 (2.8%) | 6 months | 1 |
|  |  | >1 year | 2 |
| **200,000 n= 76 (70.4%)** | Once daily n= 2 (1.9%) | 1 week | 2 |
|  | On alternate days n= 2 (1.9%) | 10 days | 1 |
|  |  | 3 weeks | 1 |
|  | Once in 5 days n= 3 (2.8%) | 2 weeks | 1 |
|  |  | 3 weeks | 1 |
|  |  | 2 months | 1 |
|  | Once a week n= 37 (34.3%) | 1 month | 5 |
|  |  | 5 weeks | 4 |
|  |  | 6 weeks | 4 |
|  |  | 7 weeks | 1 |
|  |  | 2 months | 8 |
|  |  | 3 months | 9 |
|  |  | 4 months | 2 |
|  |  | 6 months | 2 |
|  |  | 8 months | 1 |
|  |  | 10 months | 1 |
|  | Once in 10 days n= 5 (4.6%) | 2 months | 3 |
|  |  | 3 months | 1 |
|  |  | 8 months | 1 |
|  | Once in 15 days n= 19 (17.6%) | 6 weeks | 1 |
|  |  | 2 months | 4 |
|  |  | 3 months | 7 |
|  |  | 4 months | 3 |
|  |  | 6 months | 2 |
|  |  | 7 months | 1 |
|  |  | 2 year | 1 |
|  | Once in 20 days n= 1 (0.9%) | 2 months | 1 |
|  | Once a month n= 5 (4.6%) | 4 months | 1 |
|  |  | 6 months | 2 |
|  |  | 2 years | 1 |
|  |  | 9 years | 1 |
|  | Alternate days IM + oral once a week n= 1 (0.9%) | I/M for 2 weeks+ oral for 5 years | 1 |
|  | Alternate day for a week every 3 months n= 1 (0.9%) | 3 years | 1 |
| **600,000 n= 16 (14.8%)** | Once n= 2 (1.9%) |  | 2 |
|  | Once a week n= 2 (1.9%) | 1 month | 1 |
|  |  | 5 weeks | 1 |
|  | Once in 10 days n= 1 (0.9%) | 1 month | 1 |
|  | Once in 15 days n= 5 (4.6%) | 6 weeks | 1 |
|  |  | 2 months | 2 |
|  |  | 10 weeks | 1 |
|  |  | 6 months | 1 |
|  | Once a month n= 5 (4.6%) | 2 months | 1 |
|  |  | 3 months | 1 |
|  |  | 4 months | 1 |
|  |  | >1 year | 2 |
|  | Once in two and half month n= 1 (0.9%) | 10 months | 1 |
| **10,000**  **+ 200,000 n= 1 (0.9%)** | Once daily | 2 months | 1 |
|  | Once a week | 1 month |  |
| **50,000**  **+ 200,000 n= 1 (0.9%)** | Once a week | 8 months | 1 |
|  |  | 2 months |  |
| **50,000**  **+ 200,000 n= 1 (0.9%)** | Once daily | 10 days | 1 |
|  | Once in 20 days | 2 months |  |
| **200,000**  **+ 600,000 n= 1 (0.9%)** | 4 IM Injections | 3 months | 1 |
|  | 7 IM Injections |  |  |
| **200,000**  **+ 600,000 n= 1 (0.9%)** | Once a week | 6 weeks | 1 |
|  | Once |  |  |
| **200,000**  **+ 600,000 n= 1 (0.9%)** | Once in 3 months | >1 year | 1 |
|  | Once in a month |  |  |
